# A Brief Engagement Intervention Adapted for Racial and Ethnic Minority Young Adults in Mental Health Services: Protocol for a Pilot Optimization Trial

**DOI:** 10.2196/68885

**Published:** 2025-06-17

**Authors:** Kiara L Moore, Aaron H Rodwin, Marya Gwadz, Doris F Chang, Linda M Collins, Michelle R Munson

**Affiliations:** 1 Silver School of Social Work New York University New York, NY United States; 2 School of Global Public Health New York University New York, NY United States

**Keywords:** racial and ethnic minorities, mental health, young adult, engagement, optimization, adaptation, multiphase optimization strategy, factorial design

## Abstract

**Background:**

Young adults from racial and ethnic minority (REM) groups are at greater risk of disengaging from vital mental health services than their majority group peers. Emerging research suggests developmentally tailored interventions that enable personalized exploration of cultural and structural contexts; encourage trust in relationships with service providers; enhance hope for recovery; and increase self-efficacy to adhere to treatment can improve engagement among underserved REM young adults with mental health disorders.

**Objective:**

Just Do You is a brief young adult treatment engagement intervention. Although Just Do You showed evidence of efficacy with medium effect sizes in a previous clinical trial, not all participants benefited, and some of the proposed mediators were not changed. Grounded in the multiphase optimization strategy (MOST), we propose to improve the provision and effects of Just Do You by testing a set of new candidate intervention components that complement the original Just Do You program. This can potentially enhance its effects on engagement and mental health service use for REM young adults.

**Methods:**

A total of 3 new candidate behavioral intervention components and their putative mediators were designed in collaboration with REM young adults and other key informants. The new candidate components and their mechanisms of action are (1) cultural identity (perceived cultural competency), (2) future self (hope), and (3) understanding environments (self-efficacy). A pilot optimization randomized controlled trial will be conducted in an outpatient mental health setting with REM participants aged 18-34 years (N=80). The candidate components will be tested in a 2^3^-factorial design where all participants will receive Just Do You and be randomly assigned to one of 8 experimental conditions, each composed of a unique combination of candidate components. We will assess the new components’ acceptability and feasibility, and explore preliminary evidence of their effects on the primary outcome (engagement in mental health care) and mediators at a 3-month follow-up assessment.

**Results:**

The study began recruitment in December 2024 and is planned to continue through December 2026. Final study completion is planned for March 2027. The results will be disseminated via professional and scientific publications, presentations, and social media, as well as to members of the participant community.

**Conclusions:**

Findings from this study will supply critical deployment-focused evidence to inform a future randomized controlled trial as the next step in this program of research. We ultimately aim to create an effective, efficient, and scalable intervention to improve engagement and mental health outcomes among REM young adults.

**Trial Registration:**

ClinicalTrials.gov NCT06508450; https://clinicaltrials.gov/study/NCT06508450

**International Registered Report Identifier (IRRID):**

DERR1-10.2196/68885

## Introduction

### Background

In the United States, young adults (aged 18-34 years) with mental health disorders who are from racial and ethnic minority (REM) groups are at high risk for withdrawing from mental health treatment prematurely [1], particularly those with lower socioeconomic statuses [2]. Younger adults in general have lower rates of treatment for their mental health concerns than any other age group [3]. Among young adults with serious mental illness (SMI; eg, schizophrenia-spectrum and mood disorders), poor adherence to mental health care is associated with significant health and social disadvantages (eg, poverty, incarceration, and early mortality) [4,5]. On the whole, staying engaged in mental health treatment leads to improvements in health outcomes and mental health recovery [6]. However, there is very little evidence-based interventions designed for increasing the participation of young adults with mental health disorders in treatment [7], and studies show disparities in outcomes for REM groups [8,9].

### Engagement Interventions Tailored for the Transition to Adulthood

Adult mental health services are not generally equipped to address the developmental complexities of young adulthood, and typically lack services and interventions adapted specifically for young adults [10,11]. Young adulthood is an exploratory period marked by transitions and instability in various areas of life (eg, residential status, work, school, and social relationships) [12]. Treatment adherence can be a challenge under these circumstances, especially considering that young adults gain greater responsibility and autonomy over their mental health care during this developmental period [13,14]. Stopping and starting treatment is common. Because symptom onset often occurs around this age [15], deciding to drop out of treatment is risky for young adults with SMI. Without treatment, SMI symptoms can place significant constraints on developmentally appropriate exploration [16] and successful life transitions [4,17]. Recent approaches to maintaining engagement in mental health care during the transition to adulthood target individuals ranging from 15 to 35 years old [18], and have primarily focused on expanding adolescent and family service models [19-21]. However, research suggests that young people with mental disorders become less engaged with treatment once they enter adult systems of care [4,22], and that marginalized young adults may not have access to family members who can provide support [16,23].

Developmentally tailored approaches that focus on improving mental health care literacy and self-efficacy have the potential to improve engagement in treatment among REM young adults with mental health disorders [21]. Studies show REM young adults report apprehension and uncertainty about the nature of treatment and the processes for accessing it [24,25]. Research on mental health help-seeking among young adults indicates that negative perceptions about treatment play a significant role in their reluctance to initiate care [14,26]. Young adults with mental health conditions report that treatment services can seem irrelevant and unhelpful [27], and they experience stigma [28,29] and hopelessness about the future [30] that can present barriers to engagement in services [31].

### Person-Centered, Culturally Responsive Interventions

Interventions that target cultural and intersectional factors along with developmental stage are essential for engaging REM young adults living with mental health disorders [20,21,32]. The most intensive exploration of racial, ethnic, and other key social identities often happens during young adulthood [33], and informs an individual’s sense of self and direction in life [34]. REM young adults have expressed hesitancy about mental health treatment due to historical and current racism in health care [35], differences in perceived need for mental health care [36], and cultural beliefs and preferences around how to address mental health problems [37]. Studies show that REM young adults experience structural vulnerabilities (eg, living in high poverty areas, more coercive rather than voluntary treatments) [38], report low cultural competency among mental health care providers [39], along with high levels of mistrust of providers [24], which serve as key barriers to participating in treatment. Research on REM young adults in mental health care indicates that they desire greater knowledge and understanding of their identities and social positions by mental health professionals [25].

Culturally responsive interventions can improve treatment engagement by facilitating communication about the cultural and structural context from the young adult’s perspective [40]. This allows treatment providers to be responsive to young adults’ experiences and identify barriers that can arise and strengths that can be drawn upon in treatment [41-43]. Mental health interventions adapted for REM youth have incorporated culturally relevant elements (eg, language, metaphors, values, and traditions) for specific racial-ethnic groups [44]. But, adapting intervention protocols for each cultural group is not feasible for public and community mental health services that will likely provide care for youth from a wide range of backgrounds [42,45]. Also, interventions adapted for a specific racial-ethnic group may not be able to address an individual young person’s intersectional social positions (eg, gender, sexuality, wealth, immigration, and multiracial-ethnic statuses) that influence their mental health and experiences of mental health care [42]. Recent research on cultural adaptation favors person-centered approaches that invite discussion of cultural and structural factors, and allow individuals to articulate what matters most about their identities, without the need to adapt intervention protocols for prespecified aspects of cultural identity [46,47].

### Just Do You Intervention

This study tests 3 new candidate behavioral intervention components focused on cultural and structural content that can potentially be added to the Just Do You intervention in order to improve its effects for REM young adults with SMI. Just Do You [48] is an evidence-informed treatment engagement intervention that uses narrative health communication, role models, and expressive arts in a brief curriculum aimed at orienting young adults to treatment in adult mental health care settings. As young people become familiar with the staff at a clinic and the services that are available, they form impressions about treatment that allow them to make informed decisions about whether to continue. The overall goal of Just Do You is to provide an orientation to mental health treatment that will support both initial and ongoing service engagement [49]. Just Do You is delivered by a clinician and a professional peer who is further along in their mental health recovery and life experiences (a Recovery Role Model) who facilitates conversations and expressive activities based on the orientation content. This cofacilitator approach models for young adults how to collaborate with professionals in their treatment. The developmentally tailored curriculum conveys information about mental health treatment and clinic services to young adults through credible sources (eg, peer and celebrity testimonials) and multisensory activities (eg, art therapy directives) designed to hold their attention and positively shape their perceptions about treatment, stigma, and service providers [49]. In a recent randomized controlled trial (RCT) conducted at 4 sites associated with 2 large urban mental health organizations in the eastern United States (ClinicalTrials.gov, NCT03423212), Just Do You demonstrated feasibility, acceptability, and efficacy for improving treatment engagement and personal mental health recovery at 3 months after baseline, with a medium effect size, among a sample of 121 young adults with SMI, 95% of whom were from Black, Latinx, and multiracial minoritized groups [50,51].

Just Do You is based on the young adult framework of mental health service use [24]. The young adult framework was developed through studying young adults with mental health disorders and applying theories of behavior change [52,53] and mental health service use [54] to describe the phenomenon of disengagement from treatment during the transition to adulthood. Just Do You was designed to target mechanisms of engagement in services identified by the young adult framework through developmentally tailored behavior change techniques [55]. In the most recent clinical trial described above, Just Do You demonstrated statistically significant effects on four proximal mediators from the young adult framework: (1) perceived advantages of treatment, (2) trustworthiness and (3) credibility of treatment providers, and (4) stigma surrounding service use. The young adult framework was validated by trial results, but Just Do You did not impact all targeted mediators [50], including hope and self-efficacy, suggesting the need for further culturally tailored adaptations to strengthen the intervention’s influence on theoretical mechanisms of engagement and service use, as well as mental health outcomes.

### Conceptual Model

**Figure 1:**
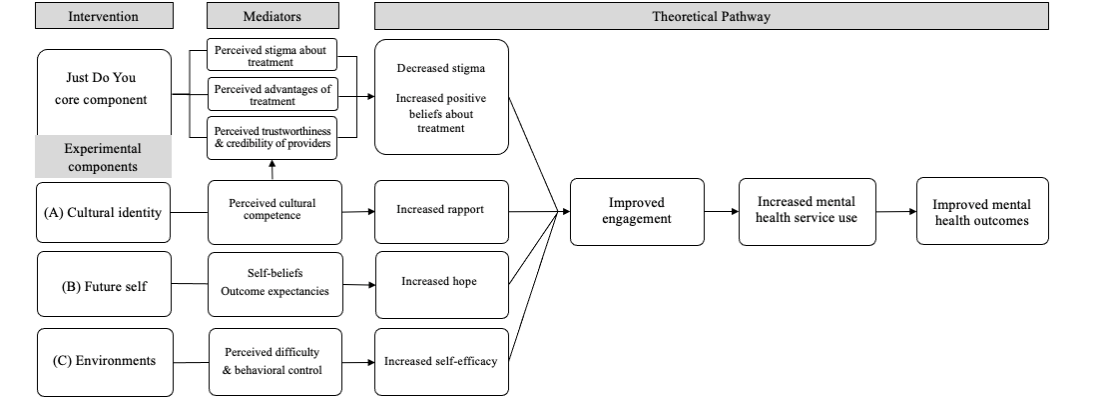
Conceptual model.

Results from the original Just Do You trial, along with findings from previous research discussed above, suggest that adding select behavioral intervention components may boost the effects of Just Do You on key mediators for REM young adults. Participant data in the original Just Do You trial across both treatment and control conditions showed that REM young adults had strong racial-ethnic identities (ie, sense of belonging and connectedness to their racial-ethnic groups) [35,56], and that strong racial-ethnic identity was associated with treatment engagement through increased hope, self-efficacy, and provider credibility ratings [57]. Building upon the conceptual model for the previous trial of Just Do You, we have designed 3 new candidate behavioral intervention components to address important mediators for REM young adults (Figure 1). The new candidate components expand upon these findings regarding the importance of racial-ethnic identities and the salience of structural barriers, and they add content to improve cultural and structural competency practices in mental health [46,58]. The new candidate behavioral intervention components seek to (1) enhance perceived cultural competency of providers, (2) improve hope (ie, self-belief and outcome expectancy that recovery is possible), and (3) improve self-efficacy for treatment engagement (ie, decrease perceived difficulty or increase control of engagement behaviors). Hope and self-efficacy were targeted as mediators in the previous trial of Just Do You but did not change [50].

### Candidate Behavioral Intervention Components

#### Overview

The new candidate components are designed to explicitly address person-centered cultural and structural content while targeting the proposed mediators of engagement specified in the conceptual model. The candidate components were selected by the first author in consultation with the research team, which included a creative arts therapist and the creator of Just Do You. All candidate components maintain the theoretical framework and delivery approach of the original intervention. The components use narratives, role models, and creative arts to preserve fidelity to the original Just Do You curriculum’s evidence-informed health communication strategies. Each candidate component was selected to meet these criteria based on preliminary research, and they were collaboratively designed using the ADAPT-ITT (Assessment, Decision, Adaptation, Production, Topical experts-integration, Training, and Testing) model [59]. ADAPT-ITT is a well-established participatory approach for modifying interventions to be effective with a specific target population. Through this process, new components were designed with REM young adults and mental health professionals, including peer service providers, in community-based adult psychiatric services. Our year-long collaborative design project produced versions of the candidate components that are more likely to be acceptable, feasible, and effective in adult community mental health settings. The 3 new candidate components, and their respective training and fidelity protocols, were manualized and formatted to complement the Just Do You core intervention as additional sessions.

#### Component A

Cultural identity is based on recent advances in cultural identity-affirming psychosocial interventions for youth [60,61] and the Cultural Formulation Interview (CFI) for *DSM-5* (*Diagnostic and Statistical Manual of Mental Disorders* [Fifth Edition]) [46]. Research suggests rapport-building strategies that prompt positive cognitions about culture in REM young adults can communicate, both implicitly and explicitly, that their cultural identities are important and will be respected within the therapeutic relationship [62]. The CFI guides mental health service providers to initiate conversations about the cultural context of individual patients at the start of treatment, in order to better address barriers that can arise and identify cultural strengths that can be drawn upon in clinical care. The CFI has been shown to enhance initial health communication and therapeutic rapport [63], and improve ongoing treatment attendance among adults and youth from minoritized racial-ethnic groups in outpatient mental health treatment [42,43].

#### Component B

Future self draws upon concepts from solution-focused brief therapy [64] and identity-based motivation in health [65] to help young adults generate positive, culturally relevant imagery of their future selves and connect those images to sustained engagement in mental health services. Solution-focused brief therapy techniques explicitly focus on generating hope and receptivity to new thoughts and actions [66]. Identity-based motivation techniques involve framing the actions needed to attain a desired future as congruent with social identities that are most salient to a young person [67]. Component B also uses elements of Photovoice [68], in which young adults take photos or videos to express a narrative storyline about their future self in recovery. Photovoice has been used as a mental health recovery intervention for people living with SMI [69,70], and previous studies highlight the utility of this approach and its alignment with the narrative and creative arts strategies in Just Do You [69,71].

#### Component C

Understanding environments draws from the structural competency framework [72] to address social and environmental factors that shape REM young adults’ engagement in mental health services (eg, unstable housing, poverty, community violence, and community protective factors). Using elements from neighborhood mapping [73,74], the professional peer facilitator (Recovery Role Model) initiates planning with young adults to manage barriers and increase their self-efficacy to sustain engagement. This approach makes use of the peer support strategy already employed in Just Do You, and previous research indicates that working with peers who relate to their social challenges is associated with greater outpatient engagement [75] and reduced disparities in service use among REM young adults with mental health disorders [76].

### Multiphase Optimization Strategy

This study will examine the new candidate intervention components using the multiphase optimization strategy (MOST) [77]. MOST is a principled method for identifying the optimal combination of intervention components before testing an intervention in a resource-intensive RCT [78]. The MOST framework consists of 3 stages: preparation, optimization, and evaluation of the intervention in a standard 2-arm RCT. This pilot trial comprises the preparation stage of MOST, in which we will pilot the study protocols to enroll and assess participants and deliver the components in a community mental health setting using a factorial design. The pilot optimization trial will establish the feasibility and acceptability of the approach and the candidate components in the intervention setting, and explore preliminary evidence of components’ effects on study outcomes and mediating variables, in preparation for a future fully powered optimization RCT.

### Study Objectives

The overall goals of our pilot research are (1) evaluate the acceptability and feasibility of the new candidate intervention components, (2) explore preliminary indications of components’ impact on engagement and service use (ie, active involvement and attendance to treatment) and on hypothesized mediators at 3-month follow-up, and (3) refine aspects of the approach for a future optimization RCT (eg, implementation, recruitment, and assessment strategies). We will test the new candidate components with REM young adult participants in an outpatient community mental health setting, using mixed methods to collect and integrate data from quantitative measures and qualitative interviews.

## Methods

### Pilot Trial Design

This study uses a balanced 2^3^ factorial experiment. Each factor will have 2 factor levels, included (yes) or excluded (no), resulting in 8 experimental conditions that represent all possible combinations of the new candidate components (see Table 1). All participants will receive the Just Do You core component, followed by the candidate components they were assigned to receive, if any. Thus, participants will be randomly assigned to one of 8 experimental conditions and receive Just Do You and 0-3 new components. We will explore preliminary evidence of component effects on the primary outcomes by comparing the mean outcomes across included conditions versus the mean outcomes across excluded conditions. For example, Component A conditions 5-8 will be compared to conditions 1-4. The factorial design does not directly compare each condition or use a traditional control group. Instead, each factor level serves as its own control. Consistent with a convergent parallel mixed methods design [79], semistructured qualitative interviews will be conducted postintervention with participants to provide context and improve our interpretation of quantitative results. A total of 80 participants will be enrolled.

**Table 1:** Experimental conditions in the 23-factorial design.

Experimental condition	Just Do You	Component A	Component B	Component C	Sample size, n
1	Yes^a^	No^b^	No	No	10
2	Yes	No	No	Yes	10
3	Yes	No	Yes	No	10
4	Yes	No	Yes	Yes	10
5	Yes	Yes	No	No	10
6	Yes	Yes	No	Yes	10
7	Yes	Yes	Yes	No	10
8	Yes	Yes	Yes	Yes	10

^a^Yes: component is included.

^b^No: component is excluded.

### Study Setting

Young adult participants will be recruited from an outpatient psychiatric rehabilitation program (the study site) in the eastern United States that serves a community of primarily adult Black and Hispanic or Latine individuals. Mental health services offered by the study site include therapeutic and skills-based group treatment, individual counseling, medication support, and resource coordination for transportation, housing, employment, and other recovery supports.

### Eligibility Criteria

Young adult participants will be eligible if they are between the ages of 18 and 34 years, identify as REM (ie, from racial and ethnic groups other than non-Hispanic, White), and are enrolled in mental health services at the study site. The eligible age range is consistent with definitions of young adulthood by the United States Census Bureau [80] and the age range most commonly targeted in early intervention services for SMI [18]. Participants will be excluded if they cannot comprehend and speak English or if they cannot understand the consent process.

### Recruitment

Participants will be recruited through flyers distributed by the research team that briefly describe the study and how to contact research staff for more information. Research staff will be present at the study site on a weekly basis to provide information in person. Interested participants who make contact with the research staff will be offered a brief eligibility screening. Participants who are eligible will be asked to provide their informed consent for enrollment.

Recruitment activities are planned to take place over 24 months. Administrative staff at the study site reported that there are about 30 young adults meeting eligibility criteria enrolled in the clinic services at any given time, with about 4 new enrollments per month. We estimate a 10% refusal rate based on the low rates of refusal in the previous Just Do You trial, which will allow us to recruit the sample over 24 months. To enhance tracking and retention for follow-up assessments, we will collect extensive contact information for all participants, which includes contact information for up to 3 individuals who will be able to locate them, and use reminder calls or texts for assessment appointments.

### Randomization

Upon enrollment in the study, participants will be assigned to an experimental condition using a randomization plan programmed into a database on the REDCap (Research Electronic Data Capture) platform [81]. The program will assign participants using block randomization to maximize balance across conditions (ie, random assignment to 1 of 8 conditions per block). Condition assignments will be unblinded.

### Intervention

The intervention period for each participant will last from 1 to 5 weeks, depending on the experimental condition. The conditions have a maximum of 5 sessions, delivered once per week, and sessions last for up to 1.5 hours (refer to content in Table 2). Following a baseline assessment, the core intervention will be delivered for all participants, and additional components will be delivered subsequently in the following weeks. In addition, participants are expected to attend all appointments for mental health treatment at the study site. All intervention components will be delivered by a mental health clinician and a professional peer specialist employed by the study site. The principal investigator will provide initial and ongoing training to the interventionists on the manualized intervention components and reporting procedures for adverse events throughout the study.

**Table 2:** Content and duration of components.

Component	Number and duration of sessions	Topics and activities
Just Do You	1 Session 60 minutes	Guiding principles of recovery Recovery Role Model personal narrative Videos of celebrity service users Personal goals related to recovery Characteristics of a trustworthy professional Introduction to clinic services
A: Cultural identity	1 Session 60 minutes	Feeling seen and heard Introduction to creative arts therapy techniques Cultural identity art activity Recovery Role Model personal narrative
B: Future self	2 Sessions 60 & 30 minutes	Session 1 Overcoming challenges and finding hope Introduction to creative arts therapy techniques Mental health resilience in music and videos Strengths and inspiration of art activity Recovery Role Model personal narrative Introduce guidelines for photo activity Session 2 Brief check-in after completing the photo activity
C: Understanding environments	1 Session 60 minutes	Overcoming barriers in the environment Introduction to creative arts therapy techniques Map of your environment art activity Recovery Role Model introduces services/strategies for addressing barriers to engagement

### Assessment Procedures

Assessments of engagement will occur at baseline, posttest (after intervention completion), and 3-month follow-up. At each assessment, participants will report on involvement in their treatment, and their perceptions of stigma, benefits, trust and rapport with providers, hopefulness, and the difficulty associated with treatment engagement. In-depth acceptability interviews will be conducted at posttest with 40 participants purposively sampled from each experimental condition. Acceptability interviews will be focused on experiences with intervention components, aspects of implementation, and any factors that promoted or impeded intervention adherence. Each assessment is expected to last for 1 hour.

Screening, consent, and assessment interviews with participants will be administered by the principal investigator or another trained member of the research team and will take place in person at the study site or via encrypted videoconferencing. All staff involved in data collection will be trained on the assessments by the principal investigator, and all research staff will complete training on informed consent, good clinical practices, and all safety and reporting protocols as certified by New York University’s institutional review board (IRB).

#### Process Evaluation

To determine the extent to which the program is delivered as intended, treatment fidelity will be monitored by research staff who observe selected sessions and rate the content delivered using checklists. Interventionists will use session checklists to record the content delivered and attendance in each session, and report on issues that arise regarding participants and study activities. The principal investigator will hold weekly supervisory meetings with interventionists to review the study process, receive feedback, and see that concerns are addressed in a timely manner.

#### Measures

Acceptability will be measured by a self-report questionnaire based on the theoretical framework of acceptability [82]. The scale has 9 items (eg, how much effort did it take you to participate in the intervention sessions?), with total scores ranging from 9 to 45, and higher scores indicating greater acceptability. Can be found in Multimedia Appendix 1 for interview guide. We will also record rates of intervention adherence as the number of assigned components completed by participants. To assess feasibility, we will record rates of recruitment, enrollment, retention, and assessment, and attempt to learn reasons for any dropouts through follow-up contacts. We will document interventionists’ feedback throughout the study to determine component challenges and facilitators, and any key aspects they believe are of particular importance to the intervention. We will also regularly meet with administrative staff from the study site to assess their perceptions of the study’s impact (challenges/benefits) on the clinic and document their ideas for overcoming any challenges to implementation. These meetings will clarify the elements involved in the feasible implementation of the intervention and the overall study, such as the integration of the study into the clinic workflow.

Engagement in treatment will be measured using 8 items from the Client Engagement in Child Protective Services Scale involvement or buy-in subscale [83]. Responses are on a 5-point Likert scale. An example item is “I am not just going through the motions. I’m really involved in working with providers.” The range for the scale is 8 to 40, with higher scores indicating higher levels of engagement. This scale has demonstrated acceptable reliability with the study target population (=.82) [51,83]. Service usage will be measured at a 3-month follow-up by participant medical records. The total number of contacts each participant makes with the treatment program, and the dates for all contacts, starting 1 month before baseline through 3-month follow-up, will be collected to assess change in service use over the study period. Demographic information will be collected at baseline, and targeted mediators and mental health symptoms will be collected by self-report measures at all timepoints. Study measures have been extensively tested by the research team [84] and previous studies with REM populations, young adults, and individuals living with SMI. All have been found to have good psychometric properties. Can be found in Multimedia Appendix 2: Baseline assessment measures for a full list of the study measures collected during the baseline assessment.

### Study Timeline

**Figure 2:**
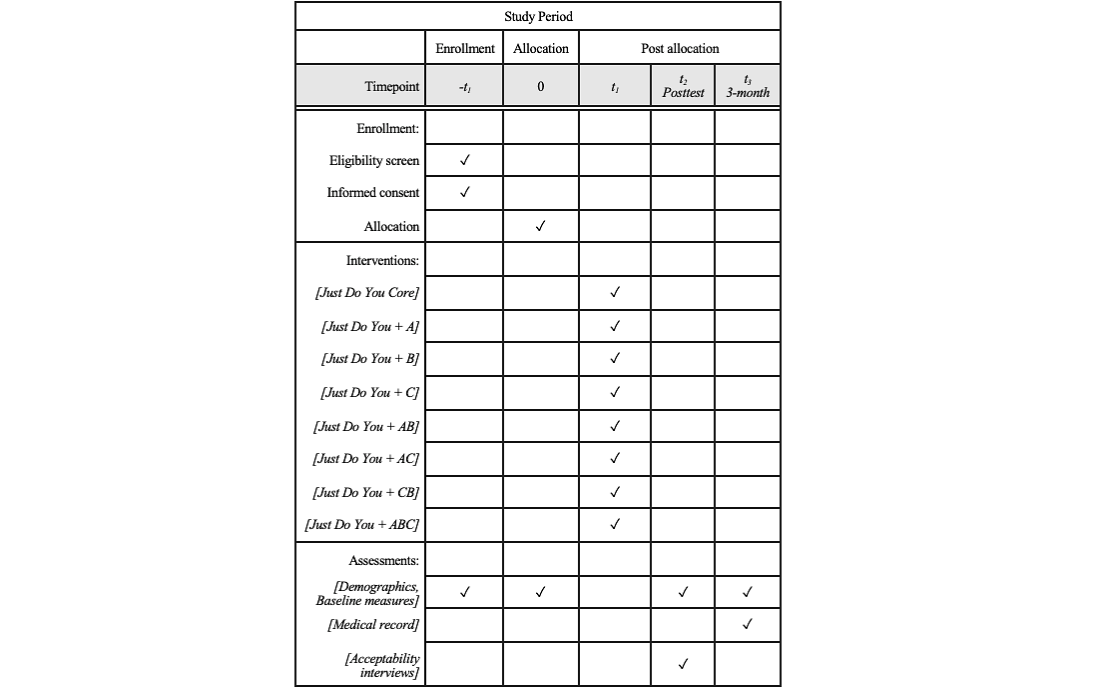
Pilot optimization trial of an engagement intervention for racial and ethnic minority young adults.

The study will take place over 4-5 months for each participant, and the total study duration is expected to last 2.5 years. Consenting participants will be enrolled, randomized, and administered a baseline assessment on the first day. They will be scheduled to receive the Just Do You core component within 1 week of completing the baseline. They will receive each subsequent component within 1 week of the previous component based on their experimental condition (Figure 2). After completing the intervention, participants will be administered the posttest assessment, which includes the study outcomes and acceptability measures. Participants will be administered the final assessment 3 months after the baseline, and the research staff will receive a report from the study site documenting each date a treatment service was received by the participant over the study period.

### Data Management

A secure REDCap database will be used to enter and manage recruitment, consent, enrollment, and assessment data. Research data will be assigned an identification code and stored separately from participant names and contact information. Only trained members of the research team will have access to the data throughout the study.

### Data Analysis

Acceptability will be based on an average rating above the mid-point of the scale and overall positive thematic qualities in the interview data. Feasibility estimates will be based on recruitment rates and retention rates for assessments and components, component adherence rates, and component fidelity rates. Benchmark criteria for feasibility will be recruitment of 3 participants per month, completed assessment rates of 75%, and components completed by 85% of participants. Quantitative data on feasibility will be summarized with descriptive statistics. Confidence intervals will convey the direction, magnitude, and precision of estimates. Infeasible study elements or elements with low acceptability will be identified and then modified or removed from the protocol before subsequent stages of intervention testing.

For qualitative data, interviews will be transcribed and analyzed to describe and assess the acceptability of the intervention components using procedures for thematic analysis [85] and coding techniques from content analysis [86]. First, analysts will code independently using a priori codes (eg, constructs assessed in quantitative measures) and determine emergent codes based on participants’ responses. Next, analysts will meet regularly to reach consensus on coding and jointly construct common, divergent, and subthemes. These processes will assist analysts to consider and manage how their own perspectives influence or guide the analytic process. Then, verbatim text will be ordered within thematic categories. Finally, data on outcomes that are assessed both qualitatively and quantitatively (eg, acceptability, feasibility, and adherence) will be integrated for side-by-side comparison using matrices and joint displays [87]. We will ensure methodological integrity by using audit trails and transparently documenting all analytic procedures and decisions [88]. Integrated results will be used to inform any future modifications to protocols and procedures for later phases of the intervention adaptation and evaluation process.

To assess treatment engagement outcomes, exploratory analyses will examine the distributions of all variables, identify outliers, and calculate descriptive statistics for each assessment timepoint, and with respect to the 3 components. We will use an intent-to-treat approach for the analysis. Factorial ANOVA using effect coding will be used to estimate the main effects and interactions between factors (ie, components) on the mean change in number of contacts with treatment services and the level of engagement at 3-month follow-up. Each intervention component will be assigned a value of +1 or –1 depending on whether it was included or excluded from the experimental condition (ie, yes or no). In addition, we will explore evidence of effects on mediators at posttest and 3-month outcomes. Analyses will rely on an examination of the overall pattern of changes (eg, strength and change in the intended direction) instead of hypothesis testing. The sample size of 80 is sufficient for the planned analyses and provides 79% power to detect main effects, corresponding to a correlation of 0.6 and a medium effect size (*d*=0.50), given α=.05.

### Ethical Considerations

The trial protocol was approved by New York University’s IRB Human Research Protection Program on December 4, 2024 (IRB-FY2024-9043). Any future protocol amendments will be reviewed by the IRB and reported on ClinicalTrials.gov. Before giving consent to enroll in the study, eligible individuals will receive a detailed description of the study and be informed that they may choose not to participate or to stop participating at any time. They will be informed of the limits to confidentiality, including that research staff will report any indications of harming themselves or others to clinical staff at the study site, and that researchers may withdraw them from the study if their health or well-being worsens.

To minimize the risk of loss of confidentiality, all informed consents and assessments will take place in a private space that assures confidential communication. Reminders of assessments made by phone or text will communicate only about the upcoming assessment appointment and not about any study or participant details. No participant information will be shared outside of the research team, with the exception of a situation where it is legally required or if the participant reports they are at risk of harm to themselves or someone else. We will follow protocols within the clinic study site and provide a “warm hand-off” to clinic staff if such a report of risk is made, or if participants require medical assistance during any research activities. While we do not foresee any event in which a participant would need to leave the study, adverse events that occur will be brought to the attention of the principal investigator and reported immediately to the IRB. Any adverse events will be reviewed and the researchers will determine whether to withdraw a participant from the study. Participants are paid US $30 in cash immediately, or by debit card in a timely manner, for each completed assessment.

### Dissemination

We will disseminate study findings in a timely fashion through presentations at scientific meetings, publications, press releases, social media, and share findings with the study site administration and participant population. Final study status will be available on ClinicalTrials.gov within one year after the study’s primary completion date.

## Results

All hiring and training of study staff for the pilot optimization trial were completed in October 2024. Participant recruitment and randomization began in December 2024 and are planned to continue through December 2026. Follow-up assessments will continue through March 2027, with final study completion (ie, data analysis and interpretation of results) planned for May 2027.

## Discussion

### Principal Findings

This study will generate the preliminary data needed for a large-scale optimization RCT of the Just Do You intervention. First, we will establish the acceptability of the new candidate intervention components when added to the existing Just Do You intervention. We anticipate that findings will show preliminary evidence of the new components’ positive impact on engagement and service use outcomes at 3-month follow-up. Second, we will ascertain the feasibility of implementing the study protocol and achieving robust recruitment, retention, adherence, and fidelity rates. These data will allow for making any necessary improvements to the components’ design or the study procedures before moving on to later stages of testing. Finally, this pilot optimization trial represents the initial stage in the MOST framework [78], which we plan to apply to improving the Just Do You intervention iteratively over time. The ultimate goal is to develop an efficient, evidence-based engagement program, tailored to be developmentally and culturally responsive to REM young adults with mental health disorders. Previous young adult mental health service engagement interventions have used RCTs to test multiple-element interventions as a treatment package [19,21], which lacks precision for determining the relative contribution of intervention components. The results of this pilot would allow us to address this gap through systematically assessing the candidate components individually in a future well-powered optimization RCT. Understanding the relative value of the candidate components is likely to inform improvements to Just Do You and lead to an optimized intervention.

Importantly, this study demonstrates a deployment-focused approach to intervention design and testing that can expedite the translation of our research into practice. The candidate intervention components were intentionally designed through incorporating the perspectives of young adults and service providers, and they are being tested in a typical community-based clinical setting. We have chosen this approach, based on previous research [48,89], to support the development of an intervention that can improve mental health service engagement and be delivered in nonacademic, real-world community treatment settings in the United States, MOST has also been used to optimize interventions, enhance their scalability, and increase the probability of successful implementation in resource-constrained settings [77]. By combining these methods, this pilot trial will allow us to collect several indicators of acceptability and fit of the approach, and determine the main constraints on implementation (eg, costs, workforce capacity, and clinic workflows). The latter will guide us in selecting an optimization objective for testing the candidate components in the future optimization RCT. We anticipate this will help to ensure that we can develop a future Just Do You intervention that is feasible, scalable, and can be disseminated widely into US community mental health services.

### Limitations

This study faces some potential challenges. First, testing an intervention with a “hard-to-engage” population often requires a considerable amount of resources. Another potential challenge is implementing a complex study with 8 experimental conditions in a clinical setting. Building on pri previous or research experience, the study team have taken several measures to ensure the success of the trial. To minimize attrition, we will use well-established methods for tracking difficult to engage populations such as on-going reminder contacts and messaging, and collecting contact information for other people we can reach respondents through. We are also allowing 2 years to recruit participants in order to have a sufficient amount of time, and to avoid overtaxing our recruitment site. In addition, we have designed the 3 new candidate intervention components to require very few additional resources to implement. Finally, the study team includes both researchers who have extensive experience with implementing MOST trials, and researchers who are skilled in conducting trials in psychiatric care settings. Our combined expertise will improve our ability to identify and address challenges in the pilot stage and determine how to enhance feasibility for future stages of the research.

### Conclusion

This research will yield critical, deployment-focused findings essential for informing a subsequent randomized controlled trial, representing the next proposed stage in this research agenda. The overarching objective is to establish an effective, efficient, and scalable intervention designed to enhance engagement and mental health outcomes within the REM young adult population.

## Appendix

Multimedia Appendix 1 Interview guide.

Multimedia Appendix 2 Baseline assessment measures.

## Abbreviations

ADAPT-ITT: Assessment, Decision, Adaptation, Production, Topical experts-integration, Training, and Testing
CFI: Cultural Formulation Interview
*DSM-5*: *Diagnostic and Statistical Manual of Mental Disorders (Fifth Edition)*
IRB: institutional review board
MOST: multiphase optimization strategy
RCT: randomized controlled trial
REDCap: Research Electronic Data Capture
REM: racial and ethnic minority
SMI: serious mental illness

## Data Availability

The datasets generated or analyzed during this study are available from the National Institute of Mental Health Data Archive on reasonable request.

## References

[1] Kresina TF, Kaplowitz L, Johnson K (2016). Human immunodeficiency virus infection in young adults: treatment of substance use disorders as a priority component of HIV prevention, care and treatment in low and middle income countries. Int J AIDS Res.

[2] Marino L, Wissow LS, Davis M, Abrams MT, Dixon LB, Slade EP (2016). Predictors of outpatient mental health clinic follow-up after hospitalization among medicaid-enrolled young adults. Early Interv Psychiatry.

[3] (2020). National Survey on Drug Use and Health, 2011 (ICPSR 34481). National Addiction & HIV Data Archive Program (NAHDAP).

[4] Lipari RN, Hedden SL (2014). Serious mental health challenges among older adolescents and young adults. The CBHSQ Report.

[5] Gralinski-Bakker J, Hauser S, Billings R, Allen J, Lyons P (2020). Transitioning to adulthood for young adults with mental health issues. Research Network on Transitions to Adulthood Policy Brief.

[6] (2019). Behavioral health: research on the costs of untreated conditions is limited (Report GAO-19-274). Government Accountability Office.

[7] Read H, Kohrt BA (2022). The history of coordinated specialty care for early intervention in psychosis in the United States: a review of effectiveness, implementation, and fidelity. Community Ment Health J.

[8] Oluwoye O, Stiles B, Monroe-DeVita M, Chwastiak L, McClellan JM, Dyck D, Cabassa LJ, McDonell MG (2018). Racial-ethnic disparities in first-Eepisode psychosis treatment outcomes from the RAISE-ETP study. Psychiatr Serv.

[9] Jones N, Kamens S, Oluwoye O, Mascayano F, Perry C, Manseau M, Compton MT (2021). Structural disadvantage and culture, race, and ethnicity in early psychosis services: international provider survey. Psychiatr Serv.

[10] (2008). Young adults with serious mental illness: some states and federal agencies are taking steps to address their transition challenges. United States Government Accountability Office.

[11] Gilmer TP, Ojeda VD, Fawley-King K, Larson B, Garcia P (2012). Change in mental health service use after offering youth-specific versus adult programs to transition-age youths. Psychiatr Serv.

[12] Meca A, Eichas K, Quintana S, Maximin BM, Ritchie RA, Madrazo VL, Harari GM, Kurtines WM (2014). Reducing identity distress: results of an identity intervention for emerging adults. Identity.

[13] Biddle L, Donovan J, Sharp D, Gunnell D (2007). Explaining non-help-seeking amongst young adults with mental distress: a dynamic interpretive model of illness behaviour. Sociol Health Illn.

[14] Gulliver A, Griffiths KM, Christensen H (2010). Perceived barriers and facilitators to mental health help-seeking in young people: a systematic review. BMC Psychiatry.

[15] McGrath JJ, Al-Hamzawi A, Alonso J, Altwaijri Y, Andrade LH, Bromet EJ, Bruffaerts R, de Almeida JMC, Chardoul S, Chiu WT, Degenhardt L, Demler OV, Ferry F, Gureje O, Haro JM, Karam EG, Karam G, Khaled SM, Kovess-Masfety V, Magno M, Medina-Mora ME, Moskalewicz J, Navarro-Mateu F, Nishi D, Plana-Ripoll O, Posada-Villa J, Rapsey C, Sampson NA, Stagnaro JC, Stein DJ, Ten Have M, Torres Y, Vladescu C, Woodruff PW, Zarkov Z, Kessler RC, WHO World Mental Health Survey Collaborators (2023). Age of onset and cumulative risk of mental disorders: a cross-national analysis of population surveys from 29 countries. Lancet Psychiatry.

[16] Institute of MedicineNational Research Council (2015). Investing in the Health and Well-Being of Young Adults.

[17] Gralinski-Baker JH, Hauser ST, Billings RL, Allen JP (2005). Risks along the road to adulthood: Challenges faced by youth with serious mental disorders. On Your Own Without A Net: The Transition to Adulthood for Vulnerable Populations.

[18] Marino L, Nossel I, Choi J, Nuechterlein K, Wang Y, Essock S, Bennett M, McNamara K, Mendon S, Dixon L (2015). The RAISE connection program for early psychosis: secondary outcomes and mediators and moderators of improvement. J Nerv Ment Dis.

[19] Kim H, Munson MR, McKay MM (2012). Engagement in mental health treatment among adolescents and young adults: A systematic review. Child Adolesc Soc Work J.

[20] Oluwoye O, Dyck D, McPherson SM, Lewis-Fernández R, Compton MT, McDonell MG, Cabassa LJ (2020). Developing and implementing a culturally informed mily otivational ngagement trategy (FAMES) to increase family engagement in first episode psychosis programs: mixed methods pilot study protocol. BMJ Open.

[21] Moore KL (2018). Mental health service engagement among underserved minority adolescents and young adults: a systematic review. J Racial Ethn Health Disparities.

[22] Manuel JI, Munson MR, Dino M, Villodas ML, Barba A, Panzer PG (2018). Aging out or continuing on? Exploring strategies to prepare marginalized youth for a transition to recovery in adulthood. Psychiatr Rehabil J.

[23] Murthy VH (2022). The mental health of minority and marginalized young people: an opportunity for action. Public Health Rep.

[24] Munson MR, Jaccard J, Smalling SE, Kim H, Werner JJ, Scott LD (2012). Static, dynamic, integrated, and contextualized: a framework for understanding mental health service utilization among young adults. Soc Sci Med.

[25] Moore KL, Lopez L, Camacho D, Munson MR (2020). A qualitative investigation of engagement in mental health services among black and hispanic LGB young adults. Psychiatr Serv.

[26] Rickwood DJ, Deane FP, Wilson CJ (2007). When and how do young people seek professional help for mental health problems?. Med J Aust.

[27] Narendorf SC, Palmer A (2016). Perception of need and receipt of mental health treatment: A three-group comparison of young adults with psychological distress. Psychiatr Serv.

[28] Yang LH, Link BG, Ben-David S, Gill KE, Girgis RR, Brucato G, Wonpat-Borja AJ, Corcoran CM (2015). Stigma related to labels and symptoms in individuals at clinical high-risk for psychosis. Schizophr Res.

[29] Rodwin AH, Shimizu R, Banya M, Moore K, Bessaha M, Pahwa R, Yanos PT, Munson MR (2025). Stigma among historically marginalized young adults with serious mental illnesses: a mixed methods study. Stigma Health.

[30] Moore KL, Camacho D, Munson MR (2020). Identity negotiation processes among Black and Latinx sexual minority young adult mental health service users. J Gay Lesbian Soc Serv.

[31] Yanos PT, DeLuca JS, Roe D, Lysaker PH (2020). The impact of illness identity on recovery from severe mental illness: a review of the evidence. Psychiatry Res.

[32] Davis B, Anglin DM, Oluwoye O, Keshavan M (2022). The unfulfilled promise of equitable first episode care for black-Americans: a way forward. Schizophr Res.

[33] Phinney JS (2006). Ethnic identity exploration in emerging adulthood. Emerging Adults in America: Coming of Age in the 21st Century.

[34] Erikson EH (1968). Identity: Youth in Crisis.

[35] Moore KL, Rodwin AH, Shimizu R, Munson MR (2024). A mixed methods study of ethnic identity and mental health recovery processes in minoritized young adults. Healthcare (Basel).

[36] Williams SL, Cabrera-Nguyen EP (2016). Impact of lifetime evaluated need on mental health service use among African American emerging adults. Cultur Divers Ethnic Minor Psychol.

[37] Chiang L, Hunter CD, Yeh CJ (2004). Coping attitudes, sources, and practices among Black and Latino college students. Adolescence.

[38] NeMoyer A, Cruz-Gonzalez M, Alvarez K, Kessler RC, Sampson NA, Green JG, Alegría Margarita (2022). Reducing racial/ethnic disparities in mental health service use among emerging adults: community-level supply factors. Ethn Health.

[39] Moore K, Camacho D, Spencer-Suarez KN (2021). A mixed-methods study of social identities in mental health care among LGBTQ young adults of color. Am J Orthopsychiatry.

[40] Bertolote J, McGorry P (2005). Early intervention and recovery for young people with early psychosis: consensus statement. Br J Psychiatry Suppl.

[41] Díaz E, Añez LM, Silva M, Paris M, Davidson L (2017). Using the cultural formulation interview to build culturally sensitive services. Psychiatr Serv.

[42] Sanchez AL, Jent J, Aggarwal NK, Chavira D, Coxe S, Garcia D, La Roche M, Comer JS (2022). Person-centered cultural assessment can improve child mental health service engagement and outcomes. J Clin Child Adolesc Psychol.

[43] Aggarwal NK, Chen D, Lam P, Lewis-Fernández R (2022). Implementing the cultural formulation interview in a community clinic to improve appointment retention: a pilot study. Psychiatr Serv.

[44] Arora PG, Parr KM, Khoo O, Lim K, Coriano V, Baker CN (2021). Cultural adaptations to youth mental health interventions: a systematic review. J Child Fam Stud.

[45] Park AL, Rith-Najarian LR, Saifan D, Gellatly R, Huey SJ, Chorpita BF (2022). Strategies for incorporating culture into psychosocial interventions for youth of color. Evid-Based Pract Child Adolesc Ment Health.

[46] Lewis-Fernández R, Aggarwal NK, Bäärnhielm S, Rohlof H, Kirmayer LJ, Weiss MG, Jadhav S, Hinton L, Alarcón RD, Bhugra D, Groen S, van Dijk R, Qureshi A, Collazos F, Rousseau C, Caballero L, Ramos M, Lu F (2014). Culture and psychiatric evaluation: operationalizing cultural formulation for DSM-5. Psychiatry.

[47] Pina AA, Polo AJ, Huey SJ (2019). Evidence-based psychosocial interventions for ethnic minority youth: the 10-year update. J Clin Child Adolesc Psychol.

[48] Munson MR, Cole A, Jaccard J, Kranke D, Farkas K, Frese FJ (2016). An engagement intervention for young adults with serious mental health conditions. J Behav Health Serv Res.

[49] Munson MR, Jaccard J (2018). Mental health service use among young adults: a communication framework for program development. Adm Policy Ment Health.

[50] Munson MR, Jaccard J, Scott LD, Moore KL, Narendorf SC, Cole AR, Shimizu R, Rodwin AH, Jenefsky N, Davis M, Gilmer T (2021). Outcomes of a metaintervention to improve treatment engagement among young adults with serious mental illnesses: application of a pilot randomized explanatory design. J Adolesc Health.

[51] Munson MR, Jaccard J, Moore KL, Rodwin AH, Shimizu R, Cole AR, Scott LD, Narendorf SC, Davis M, Gilmer T, Stanhope V (2022). Impact of a brief intervention to improve engagement in a recovery program for young adults with serious mental illness. Schizophr Res.

[52] Ajzen I (1991). The theory of planned behavior. Organ Behav Hum Decis Process.

[53] Bandura A (1977). Self-efficacy: toward a unifying theory of behavioral change. Psychol Rev.

[54] Pescosolido BA, Boyer CA, Horwitz AV, Scheid TL (1999). How do people come to use mental health services' Current knowledge and changing perspectives. A Handbook for the Study of Mental Health: Social Contexts, Theories, and Systems.

[55] Abraham C, Michie S (2008). A taxonomy of behavior change techniques used in interventions. Health Psychol.

[56] Moore K, Munson MR, Shimizu R, Rodwin AH (2022). Ethnic identity, stress, and personal recovery outcomes among young adults with serious mental health conditions. Psychiatr Rehabil J.

[57] Moore KL, Munson MR, Jaccard J (2024). Ethnic identity and mechanisms of mental health service engagement among young adults with serious mental illnesses. J Racial Ethn Health Disparities.

[58] Hansen H, Braslow J, Rohrbaugh RM (2018). From cultural to structural competency-training psychiatry residents to act on social determinants of health and institutional racism. JAMA Psychiatry.

[59] Wingood GM, DiClemente RJ (2008). The ADAPT-ITT model: a novel method of adapting evidence-based HIV interventions. J Acquir Immune Defic Syndr.

[60] Umaña-Taylor AJ (2023). Promoting adolescent adjustment by intervening in ethnic-racial identity development: opportunities for developmental prevention science and considerations for a global theory of change. Int J Behav Dev.

[61] Rajagopalan A, Vaid E, Scheinberg B, Genaro BG, Wadsworth ME (2024). Utilizing sociocultural identity to cope with poverty-related stress: methodological insights from developing a coping intervention. Identity.

[62] Mosher DK, Hook JN, Captari LE, Davis DE, DeBlaere C, Owen J (2017). Cultural humility: A therapeutic framework for engaging diverse clients. Pract Innov.

[63] Aggarwal NK, Desilva R, Nicasio AV, Boiler M, Lewis-Fernández R (2015). Does the cultural formulation interview for the fifth revision of the diagnostic and statistical manual of mental disorders (DSM-5) affect medical communication? A qualitative exploratory study from the New York site. Ethn Health.

[64] De Shazer S, Berg IK, Lipchik E, Nunnally E, Molnar A, Gingerich W, Weiner-Davis M (1986). Brief therapy: focused solution development. Fam Process.

[65] Oyserman D, Smith GC, Elmore K (2014). Identity‐based motivation: implications for health and health disparities. J Soc Issues.

[66] Franklin C, Zhang A, Froerer A, Johnson S (2017). Solution focused brief therapy: a systematic review and meta-summary of process research. J Marital Fam Ther.

[67] Oyserman D, Terry K, Bybee D (2002). A possible selves intervention to enhance school involvement. J Adolesc.

[68] Wang C, Burris MA (1994). Empowerment through photo novella: portraits of participation. Health Educ Q.

[69] Mizock L, Russinova Z, DeCastro S (2015). Recovery narrative photovoice: Feasibility of a writing and photography intervention for serious mental illnesses. Psychiatr Rehabil J.

[70] Mizock L, Russinova Z (2018). The Process of Development and Analysis of a Photovoice Mental Health Intervention.

[71] Vansteenkiste T, Morrens M, Westerhof GJ (2021). Images of recovery: A photoVoice study on visual narratives of personal recovery in persons with serious mental illness. Community Ment Health J.

[72] Hansen H, Metzl J (2019). Structural Competency in Mental Health and Medicine: A Case‐Based Approach to Treating the Social Determinants of Health.

[73] Mathis W, Cyrus K, Jordan A, Rohrbaugh R (2019). Introducing a structural competency framework for psychiatry residents: drawing your neighborhood. Acad Psychiatry.

[74] Siber-Sanderowitz S, Glasgow A, Chouake T, Beckford E, Nim A, Ozdoba A (2022). Developing a structural intervention for outpatient mental health care: mapping vulnerability and privilege. Am J Psychother.

[75] Hiller-Venegas S, Gilmer TP, Jones N, Munson MR, Ojeda VD (2022). Clients' perspectives regarding peer support providers' roles and support for client access to and use of publicly funded mental health programs serving transition-age youth in two Southern California counties. J Behav Health Serv Res.

[76] Ojeda VD, Munson MR, Jones N, Berliant E, Gilmer TP (2021). The availability of peer support and disparities in outpatient mental health service use among minority youth with serious mental illness. Adm Policy Ment Health.

[77] Collins L, Nahum-Shani I, Guastaferro K, Strayhorn J, Vanness D, Murphy S (2024). Intervention optimization: a paradigm shift and its potential implications for clinical psychology. Annu Rev Clin Psychol.

[78] Collins LM (2018). Optimization of behavioral, biobehavioral, and biomedical interventions: the multiphase optimization strategy (MOST). Statistics for Social and Behavioral Sciences (SSBS).

[79] Creswell JW, Plano Clark VL (2017). Designing and Conducting Mixed Methods Research.

[80] Vespa J (2017). The Changing Economics and Demographics of Young Adulthood: 1975-2016.

[81] Harris PA, Taylor R, Minor BL, Elliott V, Fernandez M, O'Neal L, McLeod L, Delacqua G, Delacqua F, Kirby J, Duda SN, REDCap Consortium (2019). The REDCap consortium: building an international community of software platform partners. J Biomed Inform.

[82] Sekhon M, Cartwright M, Francis JJ (2017). Acceptability of healthcare interventions: an overview of reviews and development of a theoretical framework. BMC Health Serv Res.

[83] Yatchmenoff D (2005). Measuring client engagement from the client’s perspective in nonvoluntary child protective services. Res Soc Work Pract.

[84] Munson MR, Jaccard JJ, Scott LD, Narendorf SC, Moore KL, Jenefsky N, Cole A, Davis M, Gilmer T, Shimizu R, Pleines K, Cooper K, Rodwin AH, Hylek L, Amaro A (2020). Engagement intervention versus treatment as usual for young adults with serious mental illness: a randomized pilot trial. Pilot Feasibility Stud.

[85] Braun V, Clarke V (2006). Using thematic analysis in psychology. Qualitative Research in Psychology.

[86] Hsieh H, Shannon SE (2005). Three approaches to qualitative content analysis. Qual Health Res.

[87] Guetterman TC, Fetters MD, Creswell JW (2015). Integrating quantitative and qualitative results in health science mixed methods research through joint displays. Ann Fam Med.

[88] Saldaña J (2015). The Coding Manual for Qualitative Researchers.

[89] Kane JM, Robinson DG, Schooler NR, Mueser KT, Penn DL, Rosenheck RA, Addington J, Brunette MF, Correll CU, Estroff SE, Marcy P, Robinson J, Meyer-Kalos PS, Gottlieb JD, Glynn SM, Lynde DW, Pipes R, Kurian BT, Miller AL, Azrin ST, Goldstein AB, Severe JB, Lin H, Sint KJ, John M, Heinssen RK (2016). Comprehensive versus usual community care for first-episode psychosis: 2-year outcomes from the NIMH RAISE early treatment program. Am J Psychiatry.

