# Supplementary material for: A Brief Engagement Intervention Adapted for Racial and Ethnic Minority Young Adults in Mental Health Services: Protocol for a Pilot Optimization Trial
Source: JMIR Res Protoc. 2025 Jun 17;14:e68885. doi: 10.2196/68885 (PMC12214696; doi:10.2196/68885)
Supplement: Multimedia Appendix 1 [file resprot_v14i1e68885_app1.docx]

**ACCEPTABILITY of Just Do You Components, plus QUALITATIVE INTERVIEW GUIDE**

These are questions about your experience in the Just Do You program. Please think out loud while answering these questions and tell me about why you are choosing your answer to each question.

1. How comfortable did you feel to participate in Just Do You?

1 = very uncomfortable; 2 = uncomfortable; 3 = no opinion; 4 = comfortable; 5 = very comfortable

- Which parts of Just Do You made you feel that way?
- In terms of feeling comfortable, how did Just Do You compare to other programs you’ve been in?

2. How much effort did it take you to participate in Just Do You sessions?

1 = huge effort; 2 = some extra effort; 3 = no opinion; 4 = minimal effort; 5 = no effort at all

- Which parts of Just Do You made it that way?
- In terms of the effort it took, how did Just Do You compare to other programs you’ve been in?

3. How respectful is Just Do You of people with mental health issues?

1 = very disrespectful 2 = disrespectful 3= no opinion 4 = respectful 5 = very respectful

- What parts of Just Do You made it seem that way?
- In terms of being respectful, how does Just Do You compare to other programs you’ve been in?

4. Just Do You has improved my ability to manage my mental health.

1 = Strongly disagree 2 = Disagree 3= no opinion 4 = Agree 5 = Strongly agree

- What shows you that your ability to manage your mental health has/has not improved?
- Were certain parts of Just Do You related to that? If yes, which parts?

5. Just Do You has gotten me more involved in my mental health care.

1 = Strongly disagree 2 = Disagree 3= no opinion 4 = Agree 5 = Strongly agree

- What shows you that you’re more involved/not more involved in your mental health care?

6. It is clear to me how Just Do You has improved my involvement in my mental health care.

1 = Strongly disagree 2 = Disagree 3= no opinion 4 = Agree 5 = Strongly agree

- Did certain parts of Just Do You seem related to improving/not improving your involvement in mental health care? If yes, which parts?

7. How confident did you feel that you could do what was required to participate in Just Do You sessions?

1= not at all confident; 2 = minimally confident; 3 = no opinion; 4 = somewhat confident; 5 = very confident

- What was making you feel confident/not confident that you could participate?

8. Participating in Just Do You sessions interfered with my other priorities.

1 = Strongly agree 2 = Agree 3 = no opinion 4 = Disagree 5 = Strongly disagree

- If agree, how did it interfere?

9. How acceptable was Just Do You for you?

1 = completely unacceptable; 2 = somewhat unacceptable; 3 = no opinion; 4 = somewhat acceptable; 5 = completely acceptable

- If unacceptable, what made it that way for you?

10. What did you like most about Just Do You? Why?

11. What did you like the least? Why?

12. How would you recommend we improve Just Do You for other young adults like you?
