# Supplementary material for: A Brief Engagement Intervention Adapted for Racial and Ethnic Minority Young Adults in Mental Health Services: Protocol for a Pilot Optimization Trial
Source: JMIR Res Protoc. 2025 Jun 17;14:e68885. doi: 10.2196/68885 (PMC12214696; doi:10.2196/68885)
Supplement: Multimedia Appendix 2 [file resprot_v14i1e68885_app2.docx]

| Baseline Assessment Measures | |
| --- | --- |
| Construct | Measure and Description |
| Engagement (active involvement in services) | Client Engagement in Child Protective Services Scale: This scale is altered to measure young adult engagement in mental health services. The 8-item scale will be used to measure involvement/buy-in to their treatment. Responses are on a 5-point Likert scale. This scale has demonstrated acceptable reliability with the target population (alpha=0.82).[1,2] |
| Engagement (service utilization) | Study-based measure: Attendance will be measured using a 1 self-report (“how often do you expect to attend PROS?”) and agency medical records.[3] |
| Treatment Beliefs | Study-based measure: This is a 13-item study-based measure that assesses perceptions about the advantages and disadvantages of mental health treatment; Response options are on a 5-point Likert scale. This scale has demonstrated good reliability with the target population (alpha =0.85).[4] |
| Stigma | Study-based measure (image management): This assesses perceptions related to stigma surrounding receiving mental health treatment. Response options are on a 5-point Likert scale. This scale has demonstrated acceptable reliability (alpha = 0.83).[4] |
| Credibility of Providers | Study-based measure: This is an 8-item scale assessing the extent to which mental health providers are perceived as credible. Response options are on a 5-point Likert scale. The scale demonstrated acceptable reliability (alpha=0.93)[3] |
| Trust in Providers | Group-Based Medical Mistrust Scale: This 4-item scale assesses the extent to which service users perceive their providers as trustworthy. Response options are on a 5-point Likert scale. This scale demonstrated good psychometric properties including reliability (alpha=0.92)[5,6]  Study-specific measure: This 4-item scale assesses the extent to which clients perceive their mental health providers as trustworthy. Response options are on a 8-point scale. This scale has demonstrated strong reliability (alpha=0.92)[3] |
| Cultural Competency | Iowa Cultural Understanding Assessment: This is a 25-item scale that assesses clients’ perception of cultural competence of the treatment agency and staff. Response options are on a 5-point Likert scale. This scale has demonstrated good psychometric properties.[7] |
| Hope | Recovery Assessment Scale: This is a subscale on ‘personal confidence and hope’ (5-items) which assesses one’s perceptions of hope with respect to their personal recovery. Response options are on a 5-point Likert scale. The scale has demonstrated strong reliability including in our own work (alpha = 0.93).[8,9]  Adult Hope Scale: This is a 12-item scale that assess dispositional hope, which includes factors of ‘hope agency’ and ‘hope pathways.’ Response options are on a 9-point scale. This scale has demonstrated good psychometric properties including reliability with the target population (alpha=0.75).[9,10]  Study-based measure (mental health hope): This 4-item scale assesses perceptions of hope specific to one’s mental health and recovery. Response options are on an 8-point scale. This scale has demonstrated good psychometric properties including reliability (alpha=0.79).[3,6,11] |
| Self-Efficacy | Study-based measure: This scale consists of 8-items on perceived behavioral control (adapted from Fishbein & Ajzen). Response options are on a 5-point Likert scale. This scale has demonstrated acceptable reliability (alpha = 0.76) |
| Psychiatric Symptoms | Colorado Symptom Index: This scale consists of 14-items that assess the presence and severity of psychiatric symptoms (e.g., paranoia). Response options are on a 5-point scale. This scale has demonstrated good psychometric properties including reliability (alpha = 0.91) with the target population.[9,12] |
| Ethnic Identity | Multigroup Ethnic Identity Measure: This scale consists of 12-items that assess perceptions and feelings of affirmation and belonging toward one’s ethnic group. Response options are on a 4-point Likert scale. This scale has demonstrated good psychometric properties including reliability (alpha = 0.89) with the target population.[4,9,13] |
| Discrimination | Everyday Discrimination Scale: This is a 10-item scale that assesses perceptions and experiences of everyday discrimination. Response options are on a 7-point scale. This scale has demonstrated good psychometric properties including reliability (alpha = 0.77).[14,15] |

References

1. Yatchmenoff DK. Measuring client engagement from the client’s perspective in nonvoluntary child protective services. Research on social work practice 2005;15(2):84-96. doi: https://doi.org/10.1177/1049731504271605.

2. Munson M, Jaccard J, Moore K, et al. Impact of a brief intervention to improve engagement in a recovery program for young adults with serious mental illness. Schizophrenia Research 2022. doi: 10.1016/j.schres.2022.11.008.

3. Munson MR, Jaccard JJ, Scott LD, et al. Engagement intervention versus treatment as usual for young adults with serious mental illness: a randomized pilot trial. Pilot and Feasibility Studies 2020;6(1):1-14. doi: https://doi.org/10.1186/s40814-020-00650-w.

4. Moore KL, Munson MR, Jaccard J. Ethnic Identity and Mechanisms of Mental Health Service Engagement Among Young Adults with Serious Mental Illnesses. Journal of Racial and Ethnic Health Disparities 2023:1-13. doi: 10.1007/s40615-023-01842-9.

5. Knopf AS, Krombach P, Katz AJ, Baker R, Zimet G. Measuring research mistrust in adolescents and adults: Validity and reliability of an adapted version of the Group-Based Medical Mistrust Scale. PLoS One 2021;16(1):e0245783. doi: 10.1371/journal.pone.0245783.

6. Munson MR, Jaccard J, Scott Jr LD, et al. Outcomes of a Metaintervention to Improve Treatment Engagement Among Young Adults With Serious Mental Illnesses: Application of a Pilot Randomized Explanatory Design. J. Adolesc. Health 2021;69(5):790-96. doi: 10.1016/j.jadohealth.2021.04.023.

7. Substance Abuse and Mental Health Services Administration. *Improving cultural competence. Report No.: (SMA) 14-4849*. Rockville, MD: substance abuse and mental health services administration, 2014.

8. Salzer MS, Brusilovskiy E. Advancing recovery science: reliability and validity properties of the Recovery Assessment Scale. Psychiatric Services 2014;65(4):442-53. doi: 10.1176/appi.ps.201300089.

9. Moore K, Munson MR, Shimizu R, Rodwin AH. Ethnic identity, stress, and personal recovery outcomes among young adults with serious mental health conditions. Psychiatric Rehabilitation Journal 2022. doi: 10.1037/prj0000523.

10. Snyder CR, Harris C, Anderson JR, et al. The will and the ways: development and validation of an individual-differences measure of hope. Journal of personality and social psychology 1991;60(4):570. doi: 10.1037//0022-3514.60.4.570.

11. Rodwin AH, Banya M, Shimizu R, Jaccard J, Lindsey MA, Munson MR. Childhood adversities and suicidal ideation among young adults with serious mental illnesses: The mediating roles of perceived stress, dispositional hope, and mental health hope. Journal of Affective Disorders 2025;368:820-28. doi: https://doi.org/10.1016/j.jad.2024.09.095.

12. Boothroyd RA, Chen HJ. The psychometric properties of the Colorado Symptom Index. Administration and Policy in Mental Health and Mental Health Services Research 2008;35(5):370-78. doi: 10.1007/s10488-008-0179-6.

13. Roberts RE, Phinney JS, Masse LC, Chen YR, Roberts CR, Romero A. The structure of ethnic identity of young adolescents from diverse ethnocultural groups. The Journal of Early Adolescence 1999;19(3):301-22. doi: https://doi.org/10.1177/0272431699019003001.

14. Krieger N, Smith K, Naishadham D, Hartman C, Barbeau EM. Experiences of discrimination: validity and reliability of a self-report measure for population health research on racism and health. Social science & medicine 2005;61(7):1576-96. doi: 10.1016/j.socscimed.2005.03.006.

15. Clark R, Coleman AP, Novak JD. Brief report: Initial psychometric properties of the everyday discrimination scale in black adolescents. Journal of Adolescence 2004;27(3):363-68. doi: 10.1016/j.adolescence.2003.09.004.

16. Nearchou F, Davies A, Hennessy E. Psychometric evaluation of the Multi-Dimensional Scale of Perceived Social Support in young adults with chronic health conditions. Irish journal of psychological medicine 2022;39(4):386-90. doi: 10.1017/ipm.2019.54.

17. Bruwer B, Emsley R, Kidd M, Lochner C, Seedat S. Psychometric properties of the Multidimensional Scale of Perceived Social Support in youth. Comprehensive psychiatry 2008;49(2):195-201. doi: 10.1016/j.comppsych.2007.09.002.

18. Mackenzie CS, Knox VJ, Gekoski WL, Macaulay HL. An adaptation and extension of the attitudes toward seeking professional psychological help scale 1. J. Appl. Soc. Psychol. 2004;34(11):2410-33.

19. Rodwin AH, Shimizu R, Banya M, et al. Stigma among historically marginalized young adults with serious mental illnesses: A mixed methods study. Stigma and Health 2023. doi: https://doi.org/10.1037/sah0000454.

20. Lee E-H. Review of the psychometric evidence of the perceived stress scale. Asian nursing research 2012;6(4):121-27. doi: 10.1016/j.anr.2012.08.004.
